# Reconfigurable neuromorphic memristor network for ultralow-power smart textile electronics

**DOI:** 10.1038/s41467-022-35160-1

**Published:** 2022-12-02

**Authors:** Tianyu Wang, Jialin Meng, Xufeng Zhou, Yue Liu, Zhenyu He, Qi Han, Qingxuan Li, Jiajie Yu, Zhenhai Li, Yongkai Liu, Hao Zhu, Qingqing Sun, David Wei Zhang, Peining Chen, Huisheng Peng, Lin Chen

**Affiliations:** 1 School of Microelectronics, Fudan University, 200433 Shanghai, China; 2 Zhangjiang Fudan International Innovation Center, 201203 Shanghai, China; 3 State Key Laboratory of Molecular Engineering of Polymers, Department of Macromolecular Science, and Laboratory of Advanced Materials, Fudan University, 200438 Shanghai, China

**Keywords:** Electronic devices, Electrical and electronic engineering, Synaptic plasticity

## Abstract

Neuromorphic computing memristors are attractive to construct low-power- consumption electronic textiles due to the intrinsic interwoven architecture and promising applications in wearable electronics. Developing reconfigurable fiber-based memristors is an efficient method to realize electronic textiles that capable of neuromorphic computing function. However, the previously reported artificial synapse and neuron need different materials and configurations, making it difficult to realize multiple functions in a single device. Herein, a textile memristor network of Ag/MoS_2_/HfAlO_x_/carbon nanotube with reconfigurable characteristics was reported, which can achieve both nonvolatile synaptic plasticity and volatile neuron functions. In addition, a single reconfigurable memristor can realize integrate-and-fire function, exhibiting significant advantages in reducing the complexity of neuron circuits. The firing energy consumption of fiber-based memristive neuron is 1.9 fJ/spike (femtojoule-level), which is at least three orders of magnitude lower than that of the reported biological and artificial neuron (picojoule-level). The ultralow energy consumption makes it possible to create an electronic neural network that reduces the energy consumption compared to human brain. By integrating the reconfigurable synapse, neuron and heating resistor, a smart textile system is successfully constructed for warm fabric application, providing a unique functional reconfiguration pathway toward the next-generation in-memory computing textile system.

## Abstract (web summary)

Neuromorphic computing memristors are attractive to construct low-power- consumption electronic textiles. Here, authors report an ultralow-power textile memristor network of Ag/MoS2/HfAlOx/carbon nanotube with reconfigurable characteristics and firing energy consumption of 1.9 fJ/spike.

## Introduction

As conventional complementary metal-oxide-semiconductor integrated circuits are approaching physical limits^1^, in-memory computing has emerged as an alternative low-power and high-efficiency technology^2–6^. Inspired by human brain, the basic elements of brain-inspired neuromorphic computing architectures, such as artificial neural network (ANN) and spiking neural network (SNN), are the electronic synapse and neuron^7–10^. These elements have specific nonvolatile synaptic and volatile integrate-and-fire functions, which are necessary for the system-level collaborative functioning of the neural network^11–14^. However, the fabrication processes and the materials of electronic synapses and neurons are mostly different^15^, thus causing difficulties in heterogeneous neurological integrations and limiting integration densities. Although separated artificial synapses and neurons with respective functions have been proposed for potential applications in a neuromorphic computing network, the performance mismatch between synaptic devices and neurons remains a problem to construct cooperative neural networks^16^. The need for high-efficiency cooperative neuromorphic electronics drives the development of reconfigurable memristors^17–19^. Developing reconfigurable memristor networks with functions of both artificial synapse and neurons is considered as an effective method to realize the next-generation neuromorphic electronics.

Electronic textiles with functions of displaying, sensing, energy harvesting and energy storing showed great application prospects as new-generation wearable electronics^20–22^. Integrating neuromorphic computing memristors into electronic textiles in a seamless way is crucial to efficiently store and process signals from functional electronic components^23–25^. Low energy consumption is a critical feature for wearable electronic textiles to guarantee a long working life^26^. The energy consumption of a biological neuron is at a picojoule level (pJ) with range of 1–100 pJ/spike^27–30^, which ensures that the human brain consumes extremely low energy to complete daily activities. Therefore, the fabrication of fiber-shaped reconfigurable memristor with an energy consumption lower than biological neuron has great potential in constructing ultralow-power neuromorphic computing textiles. However, typical artificial neurons are usually based on a functional circuit consisting of three electronic components such as memristors, capacitors and resistors^31, 32^, which increase the complexity and redundancy of the circuit. It remains an unmet need to achieve low-power neuron functions in simplified memristor circuits for efficient information processing in electronic textiles.

Herein, a reconfigurable memristor textile network to function as both artificial synapse and neuron with ultralow energy consumption was fabricated. By designing the heterostructure of Ag/MoS_2_/HfAlO_x_/carbon nanotube (CNT), a three-dimensional reconfigurable memristor textile network with both synapse and neuron functions was constructed, exhibiting nonvolatile resistive switching and volatile threshold-switching characteristics based on regulation of conductive filaments. In the nonvolatile mode, synaptic weights could be modulated continuously with high long-term storage capability. In the volatile mode, action potential could be inspired by information integration from prior neurons. The artificial neuron based on reconfigurable memristor not only simplifies the circuit by using a single device, but also consumes ultralow power of 1.9 fJ/spike in integrate-and-fire function. Such a power consumption is three orders of magnitude lower than that of the biological neuron. The electronic synapses and neurons based on reconfigurable memristor textile networks were integrated to realize automatic heating function as a demonstration. The ultralow-power reconfigurable neuromorphic computing textile system may open up a new avenue for bio-inspired intelligent textile electronics.

## Results

### Reconfigurable textile memristor networks

**Fig. 1:**
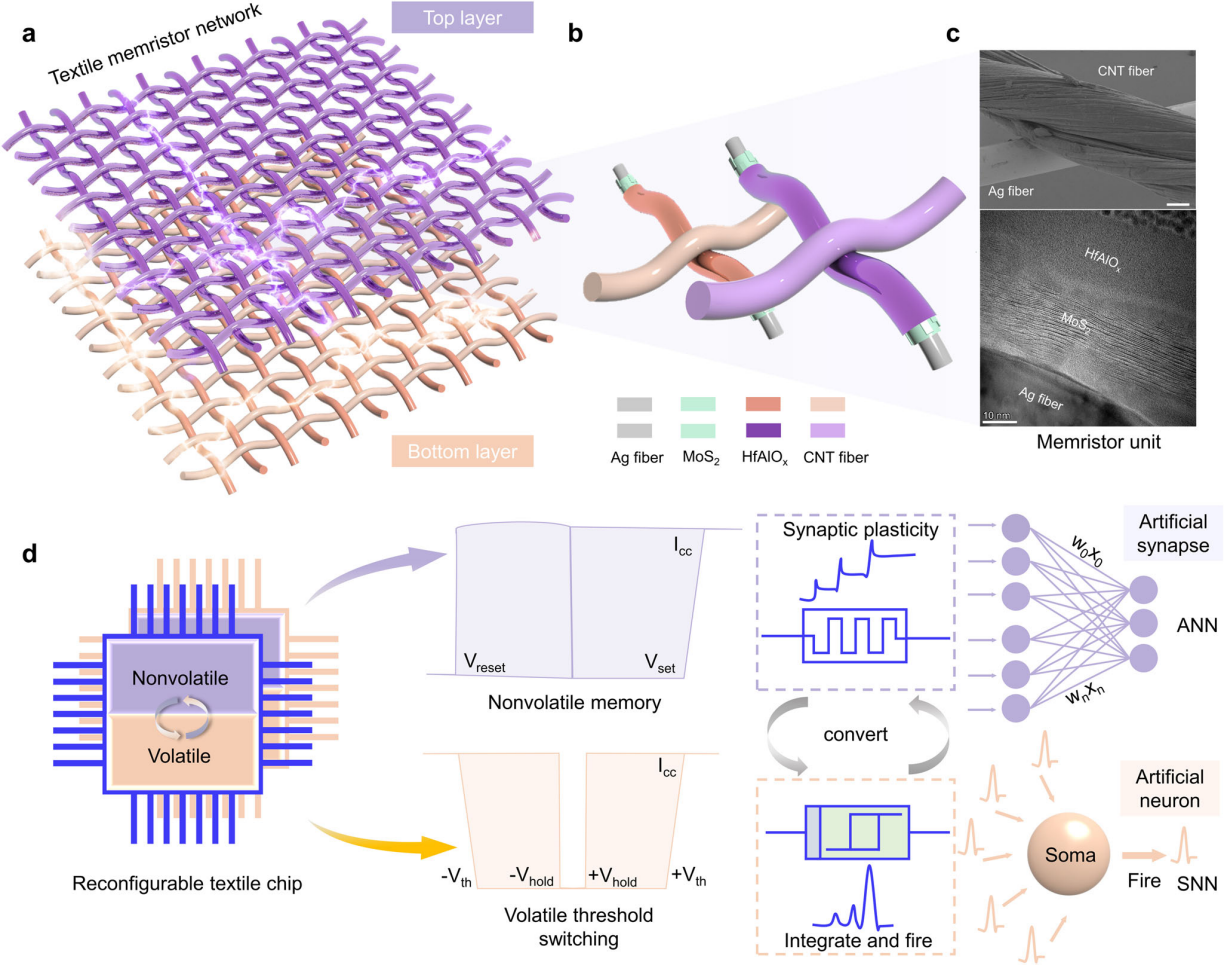
Schematic of reconfigurable textile memristor network. **a** Schematic image of textile memristor network, including top-layer device with synaptic plasticity and bottom-layer device with neural functions. **b** Structure of fiber-based memristor consisting of Ag/MoS_2_/HfAlO_x_/CNT. **c** Scanning electron microscopy (top) and cross-sectional transmission electron microscopy (bottom) images of fiber-based memristor. Scale bar of top image, 40 μm. **d** Reversible nonvolatile memory and volatile threshold-switching memristor in reconfigurable textile chip, where both artificial synaptic plasticity and integrate-and-fire functions are simulated.

To construct a wearable electronic neural network, a textile memristor was designed to simulate basic biological elements including synapses and neurons. Fig 1a illustrates a schematic of three-dimensional textile neural network made of reconfigurable textile memristors, including top and bottom layers with an interwoven structure that acts as artificial synapses and neurons, respectively. The basic reconfigurable memristor unit consists of a heterostructure of MoS_2_ nanosheets and HfAlO_x_ film. Such a structure was constructed via electrophoretic deposition and atomic layer deposition (Fig. 1b and c, Supplementary Figs. 1 and 2), providing the foundation for low-power consumption of the device. The top and bottom electrodes were made from Ag and CNT fibers, respectively. The energy-dispersive X-ray spectroscopy result and cross-sectional scanning electron microscopy images show that MoS_2_ and HfAlO_x_ heterogeneous films were evenly distributed on the Ag fiber electrode (Supplementary Figs. 3–6).shows the reconfigurable textile network with nonvolatile memory and volatile threshold-switching characteristics, which can switch between the two modes via controlling compliance current. The top-layer textile memristor with a nonvolatile behavior acts as artificial synapse, which is the core component for ANN-based neuromorphic computing. The soma of post-neuron can effectively integrate diverse information acquired from different pre-neurons and form the next-level action potential in SNN, which is emulated by the reconfigurable bottom-layer memristor. The reconfigurable textile network with the same basic units enables the effective matching between different functional units (artificial synapse and neurons), thus enhancing the unity of preparation process and reducing the difficulty of integrating neuromorphic computing networks. These features make the entire electronic neural network more flexible and adaptable to the multiple-change application scenarios and calculation requirements.

### Artificial synapse emulated by nonvolatile memory

**Fig. 2:**
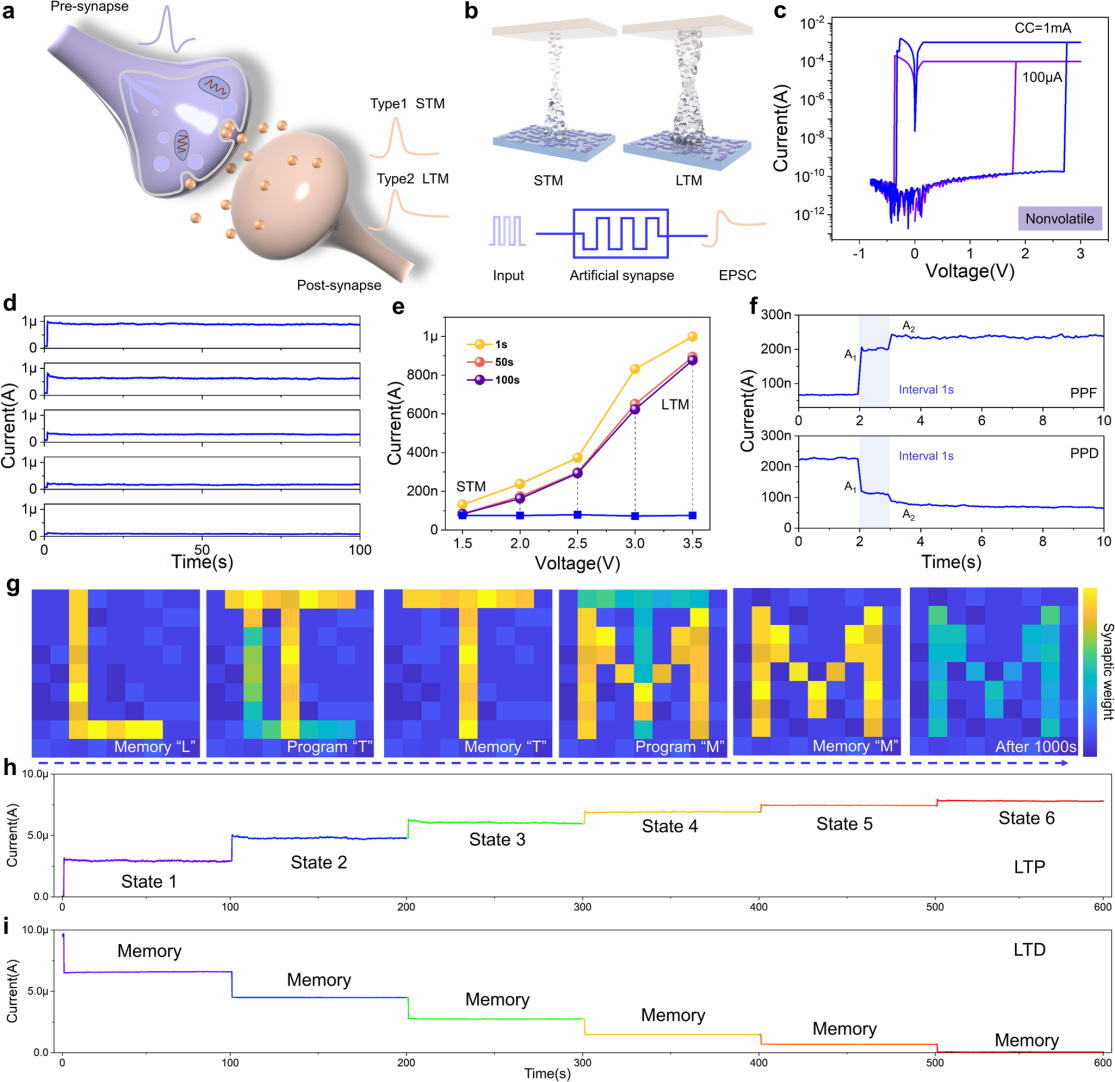
Nonvolatile storage characteristic of artificial synapse. **a** Schematic of biological synapse, which could be emulated by artificial synapses with short-term and long-term plasticity. **b** Schematic of switching mechanism for short-term memory (STM) and long-term memory (LTM) in artificial synapse. Excitatory post-synaptic current (EPSC) could be inspired by electrical pulse applied to device. **c** Nonvolatile resistive switching curves with compliance current (CC) of 100 μA and 1 mA. **d** EPSC behaviors of artificial synapse under different electrical pulse amplitudes, including 1.5, 2, 2.5, 3, and 3.5 V. **e** Device transition from STM to LTM as a result of enhanced pulse amplitude, extracted from panel **d**. **f** Paired pulse facilitation (PPF) and paired pulse depression (PPD) characteristics generated by a pair of pulses with an interval of 1 s. **g** Real-time program and storage for letters of “L”, “T” and “M” based on synaptic weights of memristor array. **h**, **i** Long-term potentiation (LTP) and long-term depression (LTD) curves of artificial synapses with multi-level storage states, respectively.

The plasticity of bio-synapse could be emulated by applying pulses to pre-terminal of artificial synapses (Fig. 2a). The short or long-term memory characteristics are defined by the maintained time of changed synaptic weighs^33–35^, corresponding to the weak and strong conductive filaments in the memristor (Fig. 2b). Nonvolatile resistive switching curves of the devices were achieved by setting compliance currents of 100 μA and 1 mA (Fig. 2c), which enable the artificial synapse to store multiple synaptic weight values. Both endurance and retention (10^4^ s) with a high on/off ratio of ~10^6^ were achieved for realizing a reliable nonvolatile memory, as shown in Supplementary Figs. 7 and 8. For the wearable application of device, nonvolatile resistive switching characteristics were experimentally demonstrated under different straining states (Supplementary Figs. 9 and 10). The uniform distribution of both high and low resistance states among 30 different devices further proved the reliability of synaptic memristors for array-level applications (Supplementary Figs. 11 and 12).

During the simulation of excitatory post-synaptic current, the current of memristor increased when the amplitudes of the single electrical pulses (pulse width of 10 ms) increased from 1.5, 2, 2.5, and 3 to 3.5 V (Supplementary Fig. 13), indicating the transformation from short to long-term memory of artificial synapse (Fig. 2d and e). When the pulse number increased to a pair of pre-spikes, paired pulse facilitation and depression with an interval of 1 s were triggered, which could be fitted using a double exponential decay function (Fig. 2f and Supplementary Fig. 14). Array-level long-term memory was further demonstrated upon application of electrical pulses (pulse width of 50 ms, amplitude of 3 V) on specific units of textile memristor network, as shown in Fig. 2g. The letters “L”, “T”, and “M” were input into a 9 × 9 array for real-time 81-pixel images, which could be maintained for 1000 s until refresh operations were carried out with negative pulses (pulse width of 50 ms, amplitude of –2.5 V), as shown in Supplementary Fig. 15. These results are pertaining to programming and erasing processes, indicating that all measured devices showed comparable and uniform performances. As the number of electrical stimuli increased, the modulation on multiple synaptic weights including long-term potentiation and depression were verified in Fig. 2h and i, which are important for training accuracy in ANN-based pattern recognition^36–38^. There were six-level conductance states with stable retention behaviors over 100 s under a small bias of 100 mV (Supplementary Fig. 16), which might be attributed to strong conductive filaments in the active layer of textile memristor.

### Ultralow-power artificial neuron

**Fig. 3:**
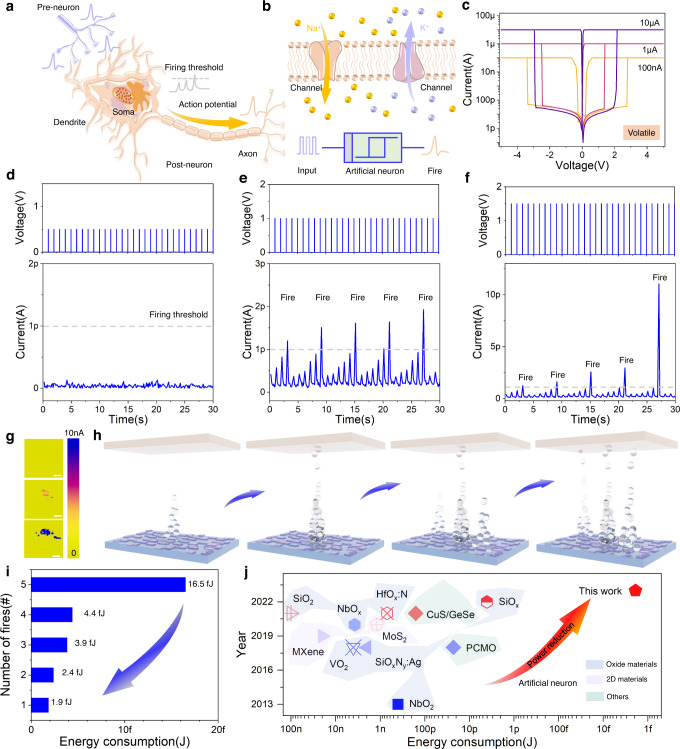
Volatile characteristic of ultralow-power artificial neuron. **a** Schematic of biological neuron with a core integrate-and-fire function. **b** Dynamic membrane potential generation process based on ion channel. **c** Volatile resistive switching curves under different compliance currents of 100 nA, 1 μA, and 10 μA. **d** Current-time curve of volatile device under s mall pulses (0.5 V, 1 ms) without response. The fire threshold is 1 pA. **e** Integrate-and-fire function realized by applying pulses of 1 V/ 1 ms. **f** Integrate-and-fire function with enhanced response under pulses of 1.5 V/ 1 ms. **g** Conductive atomic force microscopy surface map of Ag/MoS_2_/HfAlO_x_ film under different scanning voltage values of 0, 5, and 10 V. Scale bar, 200 nm. **h** Schematics of integrate-and-fire behavior of memristive neuron under pulses. **i** Calculated artificial neuron power under continuous pulses of 1.5 V/1 ms. **j** Comparison of firing energy consumption in recently reported artificial neurons.

Neurons consisting of soma, dendrites and axons are necessary for information integration and transmission^39^, where the action potentials could be induced via ion diffusion inside and outside of cell membrane (Fig. 3a and b). A typical artificial neuron needs at least three kinds of electronic components to form a working circuit for simulating integrate-and-fire function, as shown in Supplementary Fig. 17. The reconfigurable fiber-shaped memristor provides a new pathway for constructing a single device-based artificial neuron, which is based on the natural characteristic of pulse signal integration of the device. By designing weak conductive filaments with compliance currents of 10 μA, 1 μA, and 100 nA, the reconfigurable memristor exhibited volatile threshold-switching behaviors and could be reconstructed from artificial synapse to artificial neuron (Fig. 3c).

The integrate-and-fire function with features of all-or-nothing, strength-modulated frequency response and threshold-driven spiking were simulated by applying consecutive electrical pulses to the artificial neuron (pulse width of 1 ms, amplitudes of 0.5, 1, and 1.5 V), where the firing threshold was set as 1 pA, as shown in Fig. 3d–f, Supplementary Fig. 18. Conductive atomic force microscopy was employed to analyze the current mapping image and working mechanism of the memristive neuron (Fig. 3g, Supplementary Fig. 19). Current value and area in the film of MoS_2_/HfAlO_x_ gradually increased when the voltage applied on the top electrode of Ag fiber, indicating that the conductive filaments gradually grew and dispersed into multiple channels. Besides, the electrical threshold-switching curve under positive voltage was fitted using models of Ohm’s law, trap and trap-filled limited space charge limited current. The fitting results were shown in Supplementary Fig. 20. The mechanism of reconfigurable characteristics in device could be attributed to the modulation of Ag conductive filaments in functional layer, where weak and strong conductive filaments were formed in volatile and nonvolatile switching states of device, respectively (Supplementary Figs. 21, 22). Moreover, such results further support the mechanism diagrams shown in Fig. 3h.

Energy consumption is one of the most important parameters used to measure the performance of an artificial neuron. Inspired by biological neuron, the energy consumption of artificial neuron device was defined as the power value of a single spike in integrate-and-fire behavior. Figure 3i shows the firing energy consumption (E_*firing*_) of Ag/MoS_2_/HfAlO_x_/CNT based memristive neuron, which can be reduced to 1.9 fJ/spike calculated by the formula E_*firing*_ = V × I_*firing*_ × t. Compared with the energy consumption (pJ level) of the state-of-art artificial neurons based on various materials, including oxide materials^32, 40–45^, two-dimensional materials^46, 47^, and others^48, 49^, the fabric artificial neuron based on MoS_2_/HfAlO_x_ heterostructure shows obvious competitiveness and advantages (Fig. 3j, Supplementary Figs. 23, 24). The energy consumption of the memristive neuron proposed in this work is at least three orders of magnitude lower than that of biological neuron, exhibiting a high application potential in ultralow-power neuromorphic computing textile network.

### Integration of smart heating textile system

**Fig. 4:**
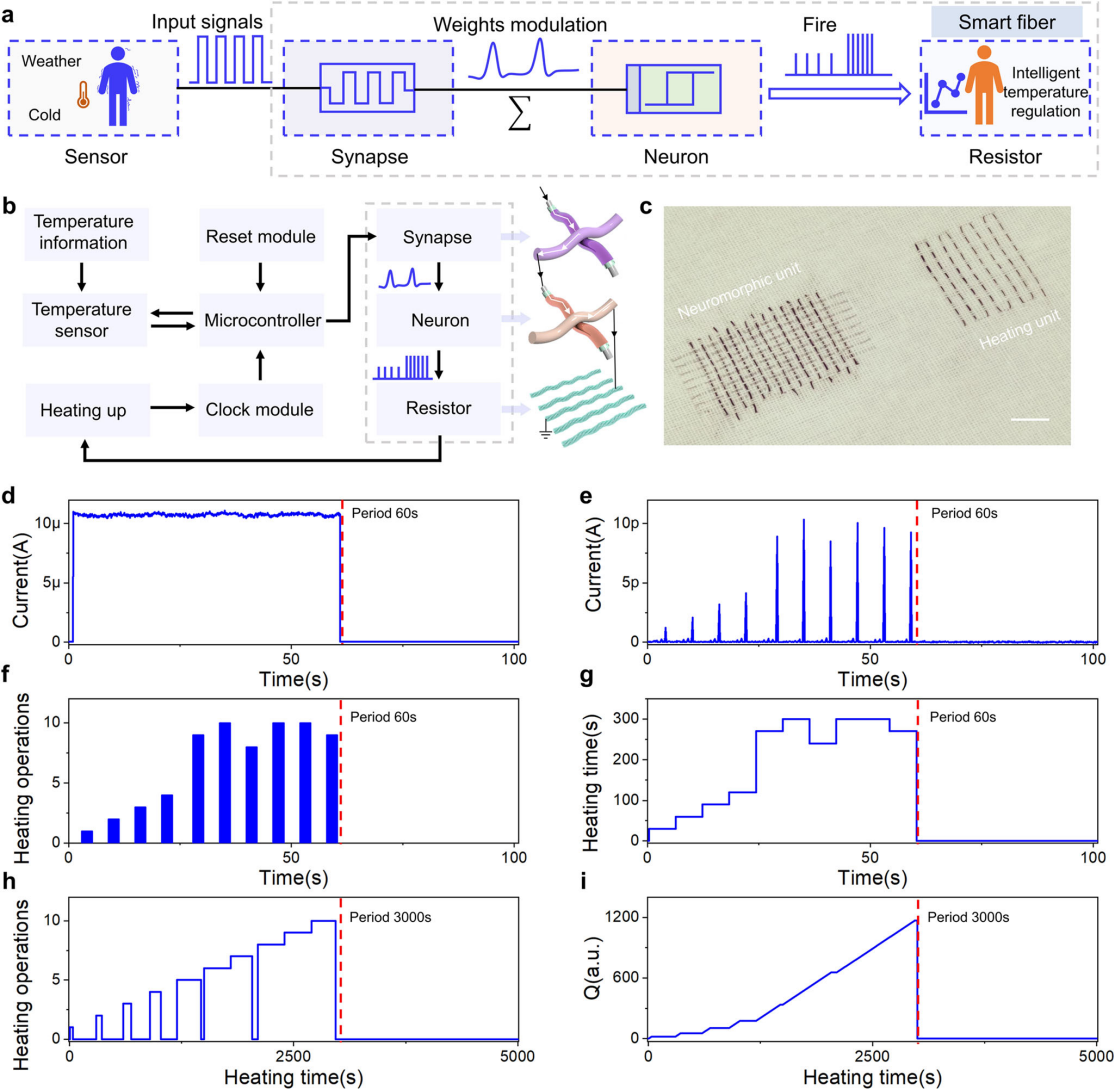
Intelligent heating textile based on reconfigurable memristor. **a** Schematic of intelligent fiber heating system. The key fiber units are artificial synapse, artificial neuron and resistor. **b** Operation mechanism of the intelligent warm fiber. **c** Photograph of the fiber-based intelligent heating memristors. Scale bar, 1 cm. **d** Current-time curve of artificial synaptic device, which could inspire the connected artificial neuron. After a period of 60 s, the device was initialized for the next cycle. **e** Current-time curve of artificial neuron based on the signal of synaptic device. **f** Heating operations of fiber-based resistor inspired by the signal of memristive neuron. **g** Calculated heating time corresponding to the plot in panel **f**. **h** Heating time curve of resistor for real heating operation. **i** Released resistor heat versus heating time. The period of heating resistor was 3000 s.

In biological systems, synapses are connected to neurons to build complete neural network and realizing complex life activities. Inspired by collaborative working mode of three-dimensional neural network^50^, an intelligent heating textile system was designed by integrating fabric synapse, neurons and heating resistors (Fig. 4a, Supplementary Fig. 25). The block diagram of the intelligent heating system shows that the functional fibers of artificial synapse, neurons and resistors (marked in dotted line in Fig. 4b) were connected sequentially, which plays key roles in modulation of synaptic weights, integrate-and-fire and heating up, respectively. The frequency and times of heating operations with a period of 60 s was regulated by artificial synapse and neurons, which acted as neuromorphic units. The resistors acting as a heating unit executed the heating operations (Q = I^2^ × R × t) with a period of 3000 s, as shown in Fig. 4c.

When the environment temperature was low and identified as cold, the input pulses could be applied to artificial synapse in order to modulate weights (Fig. 4d). The increased conductance of synaptic device could decrease the voltage division, further increasing the voltage division of series artificial neurons. Due to the threshold-driven spiking feature of the neuron, pulses with increased amplitude could induce firing responses with different values. These responses could be converted into time and period for heating operation, as shown in Fig. 4e–g. The heating information could be subsequently transferred to resistors for releasing heat to increase the temperature of textile system (Fig. 4h and i). When the period threshold was reached, the system was initialized to further determine whether the next heating cycle should be initiated. The intelligent heating textile system provides an alternative candidate for next-generation smart clothes and wearable electronics.

## Discussion

In summary, we present a functional textile network consisting of reconfigurable memristors, which was based on the structure of Ag/MoS_2_/HfAlO_x_/CNT with nonvolatile memory and volatile threshold-switching characteristics. Multi-level conductance modulation was achieved by the artificial synapse of top layer in the textile network. Integrate-and-fire function was simulated by the reconfigurable neuron of bottom layer in the textile network, which showed ultralow energy consumption of 1.9 fJ/spike, at least three orders of magnitude lower than that of biological neurons and reported artificial neurons. The artificial synapse, neuron and functional resistor were integrated into a heating textile system for intelligent temperature modulation. The ultralow-power textile memristor network could provide new directions in the development of brain-inspired reconfigurable and wearable neuromorphic computing electronics for intelligent Internet of Things applications.

## Methods

### Device fabrication

The memristor network was fabricated using the Ag fiber for the top electrode, which was sequentially cleaned by acetone, isopropanol and deionized water each for five minutes. Then, the MoS_2_ nanosheet film was deposited on the Ag fiber by electrophoretic deposition based on ethanol solvent. Subsequently, 20 nm oxide layer of HfAlO_x_ was deposited on the fiber at 130 °C via atomic layer deposition. The atomic layer deposition process consisted of one cycle trimethylaluminum, one cycle H_2_O, one cycle tetrakis(ethylmethylamino)hafnium and one cycle H_2_O. The carrier gas used between different steps was Ar. Lastly, the reconfigurable memristor was fabricated by interlacing Ag fiber with the deposited film and CNT fiber.

CNT fiber was spun from a CNT array synthesized by chemical vapor deposition at 1250 °C. Ethanol/acetone and ferrocene were used as the carbon source and catalyst, respectively. The spinnable CNT acted as resistors for heating in the smart system without any additional processing.

### Device characterization

Ag/MoS_2_/HfAlO_x_/CNT architecture was studied by ZEISS SIGMA HD field emission scanning electron microscope. The sectional view of the memristor was acquired by transmission electron microscopy (Talos-F200). The maps of current filaments were measured using C-AFM (XE-100) for studying the working mechanism of the memristor. The direct-current scanning measurements of memristor were conducted by Agilent B1500A. Electrical pulse tests were carried out via Semiconductor Pulse Generator Unit and waveform generator/fast measurement unit in air atmosphere.

## Supplementary information

Supplementary Information

## Data Availability

All relevant data are available within the article and Supplementary Information, or available from the corresponding authors upon reasonable request.

## Code availability

All codes used in simulations supporting this article are available from the corresponding authors upon reasonable request.
