# Supplementary material for: Reconfigurable neuromorphic memristor network for ultralow-power smart textile electronics
Source: Nat Commun. 2022 Dec 2;13:7432. doi: 10.1038/s41467-022-35160-1 (PMC9718838; doi:10.1038/s41467-022-35160-1)
Supplement: Supplementary file 1 — Supplementary Information [file 41467_2022_35160_MOESM1_ESM.pdf]

Supplementary Information for

Reconfigurable neuromorphic memristor network for ultralow-power smart textile electronics

Tianyu Wang[1,#], Jialin Meng[1,#], Xufeng Zhou[2,#], Yue Liu[2], Zhenyu He[1], Qi Han[1], Qingxuan Li[1], Jiajie Yu[1], Zhenhai Li[1], Yongkai Liu[1], Hao Zhu[1], Qingqing Sun[1], David Wei Zhang[1], Peining Chen[2*], Huisheng Peng[2] and Lin Chen[1*]

[1]School of Microelectronics, Fudan University, Shanghai 200433, China; Zhangjiang Fudan International Innovation Center, Shanghai 201203, China.

[2]State Key Laboratory of Molecular Engineering of Polymers, Department of Macromolecular Science, and Laboratory of Advanced Materials, Fudan University, Shanghai 200438, China.

[#]These authors contributed equally to this work.

*Correspondence and requests for materials should be addressed to Peining Chen (peiningc@fudan.edu.cn), and Lin Chen (linchen@fudan.edu.cn).

This PDF file includes:

Supplementary Figures 1 to 25 (Pages S2-S26)
Supplementary References (Page S27)

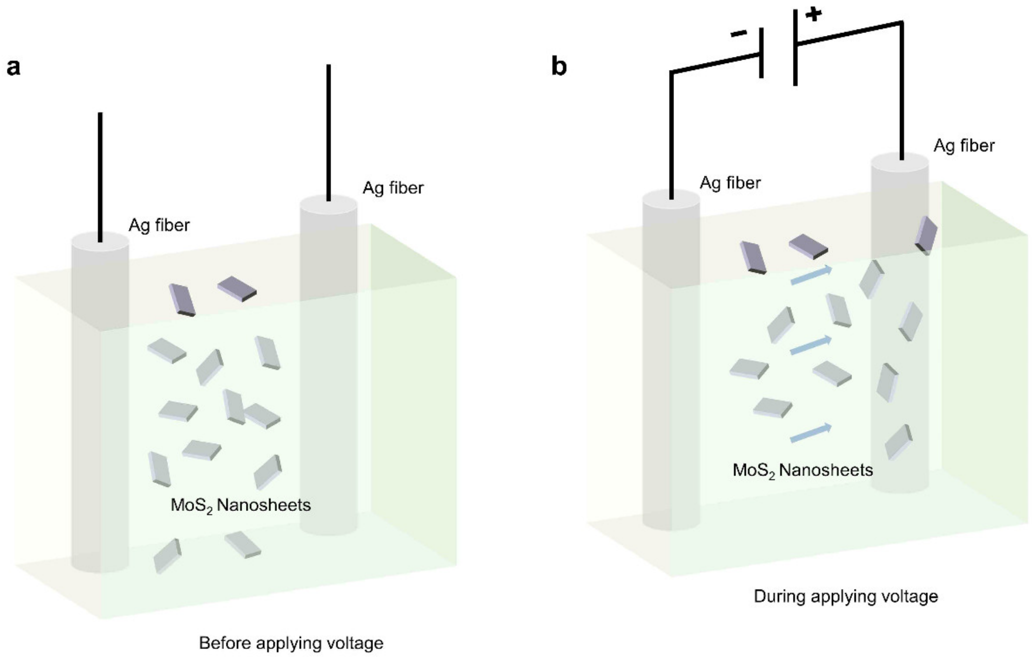

Supplementary Fig. 1 | The schematic of electrophoretic deposition method used for growing MoS_2 film on Ag fiber (a) before applying voltage and (b) during applying voltage. The MoS_2 nanosheets could be deposited on the anode of Ag fiber by electrophoretic method[1].

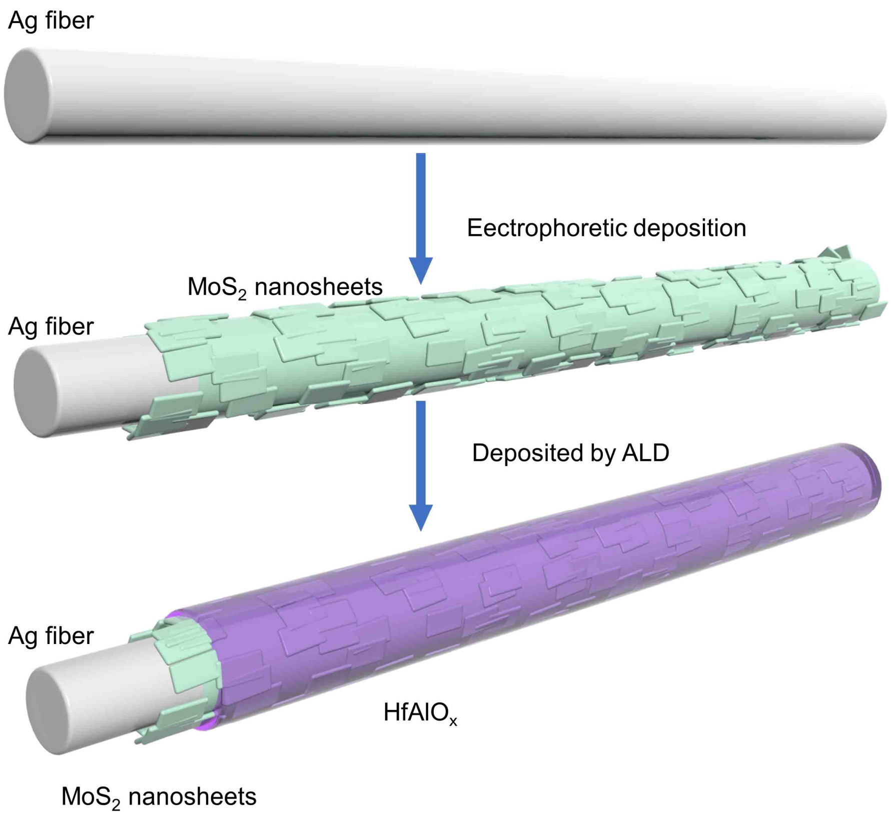

Supplementary Fig. 2 | The fabrication process of fiber-based memristor. Firstly, MoS_2 nanosheets film was deposited on Ag fiber by electrophoretic deposition method. Then atomic layer deposition (ALD) was used to grow $HfAlO_x$ film on the MoS_2 deposited Ag fiber at 130 °C, which could form complete device by interlacing this fiber and CNT fiber.

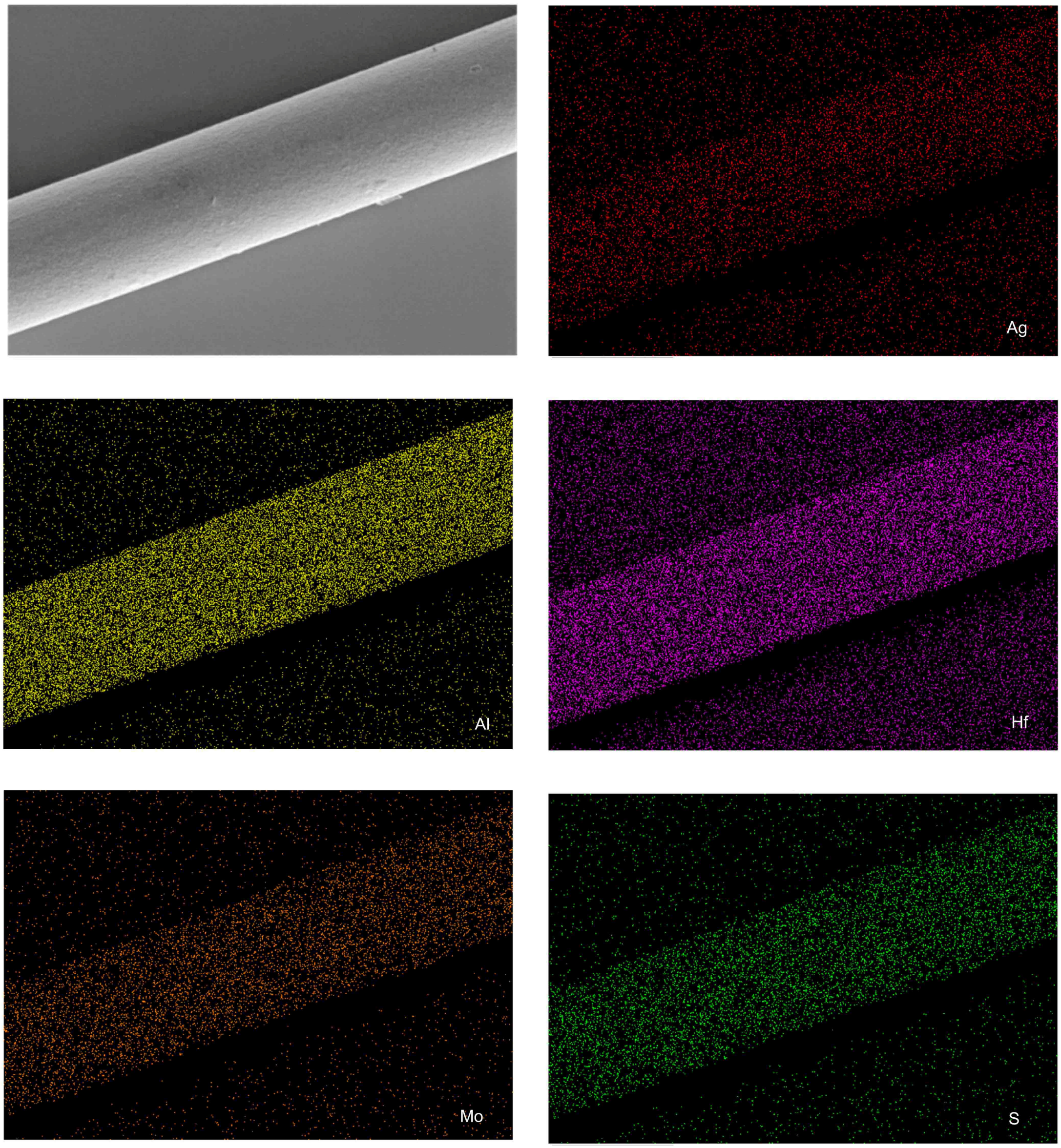

Supplementary Fig. 3 | Scanning electron microscopy (SEM) image and energy-dispersive X-ray spectroscopy mapping of the Ag fiber with MoS_2 and $HfAlO_x$ films, where the elements of Ag, Mo, S, Al and Hf are distributed uniform, indicating the high-quality preparation method of electrophoretic deposition and ALD.

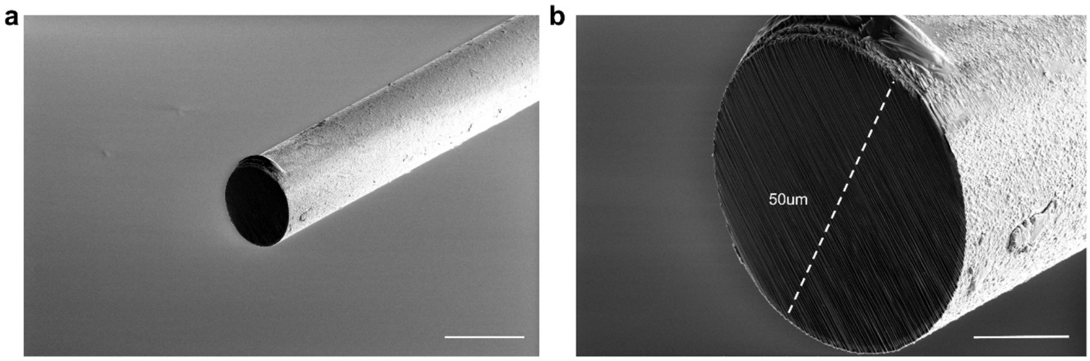

Supplementary Fig. 4 | The cross-sectional SEM image of Ag fiber with scale bar of a) 50 μm, and b) 20 μm, showing that the diameter of Ag fiber is 50 μm.

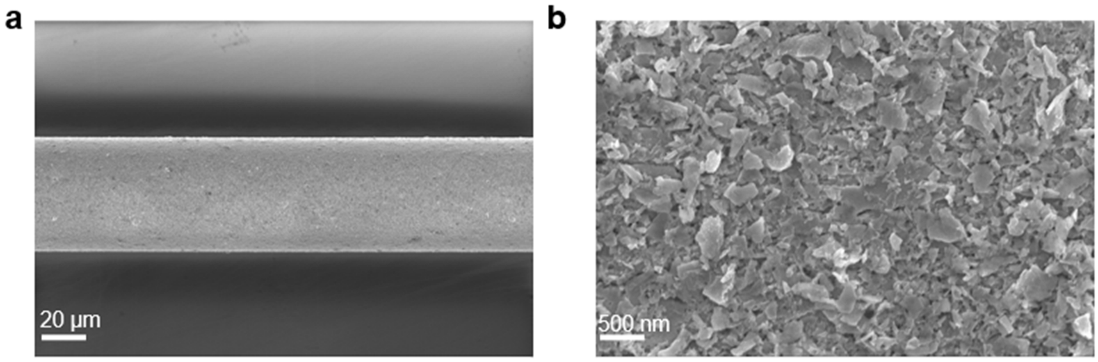

Supplementary Fig. 5 | SEM images of MoS_2 nanosheets grown on Ag fiber with scale bar of a) 20 µm, and b) 500 nm.

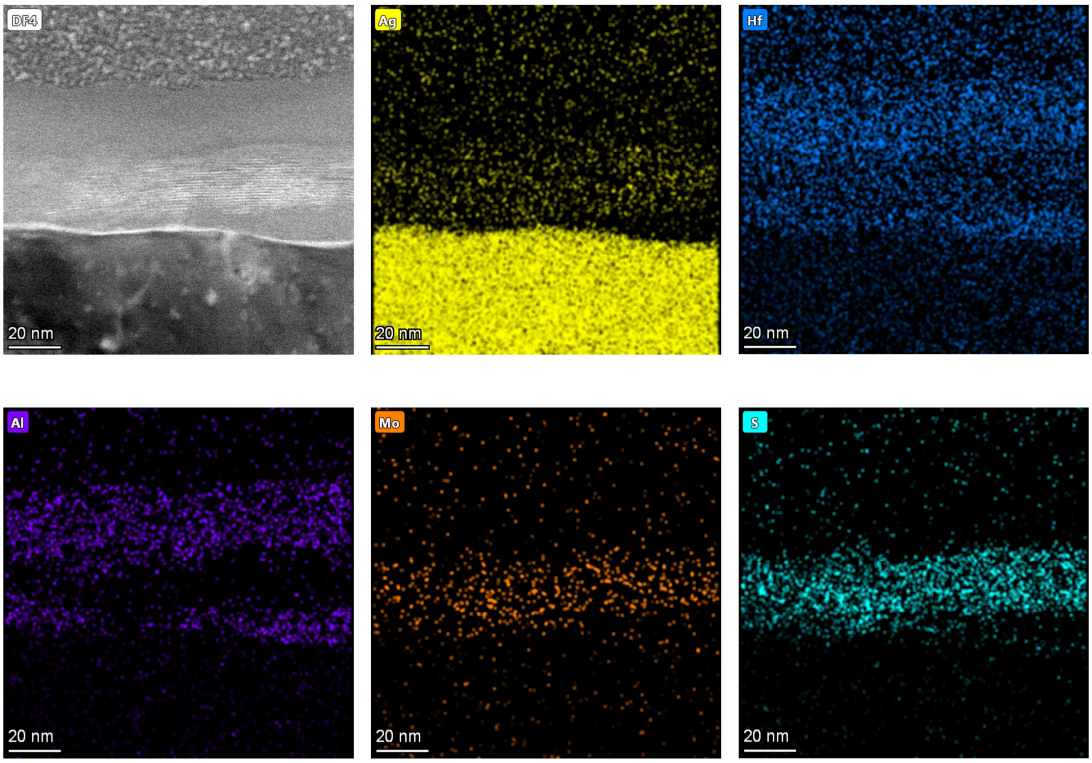

Supplementary Fig. 6 | Cross-sectional transmission electron microscopy (TEM) image and energy-dispersive X-ray spectrometry elemental mappings of heterostructure of MoS_2 and $HfAlO_x$ films. The MoS_2 nanosheets and $HfAlO_x$ film are closely combined due to the excellent step coverage of ALD.

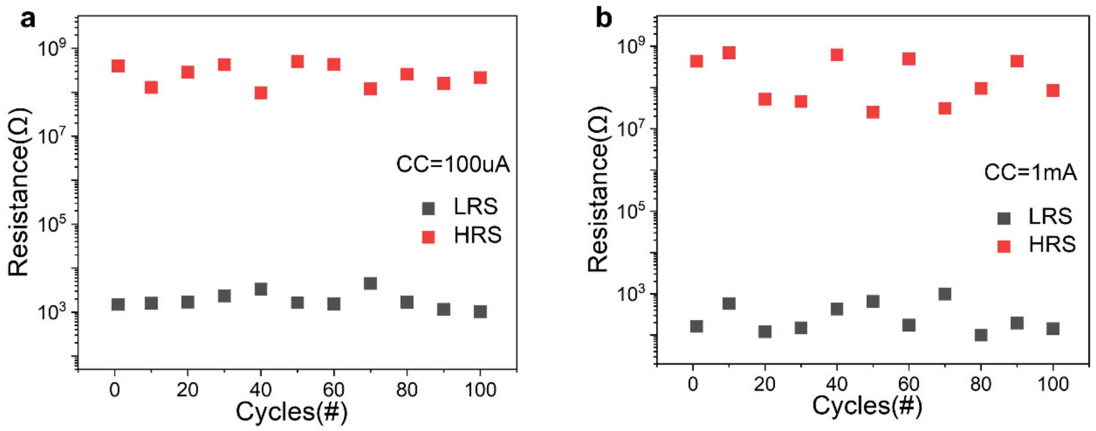

Supplementary Fig. 7 | Endurance characteristics of nonvolatile memristors under different compliance currents. (a) Under compliance current (CC) of 100 μA, the high resistance state (HRS) and low resistance state (LRS) switched continuously over 100 cycles. (b) Under compliance current of 1 mA, the memristor exhibited on/off ratio of ~10^6 during 100 cycles of switching operation.

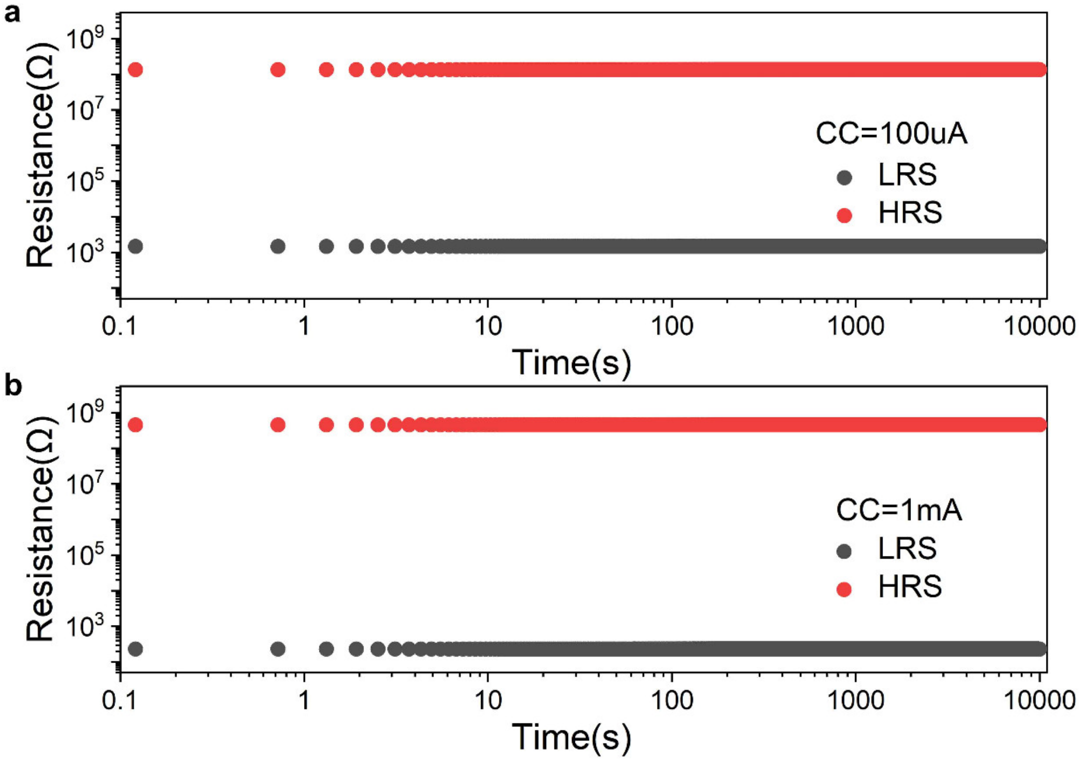

Supplementary Fig. 8 | The retention curves of nonvolatile memristors under different compliance currents. (a) Under CC of 100 μA, the memristor exhibited reliable retention characteristic with HRS and LRS over 10,000 s. (b) Under CC of 1 mA, HRS and LRS could be well maintained over 10,000 s.

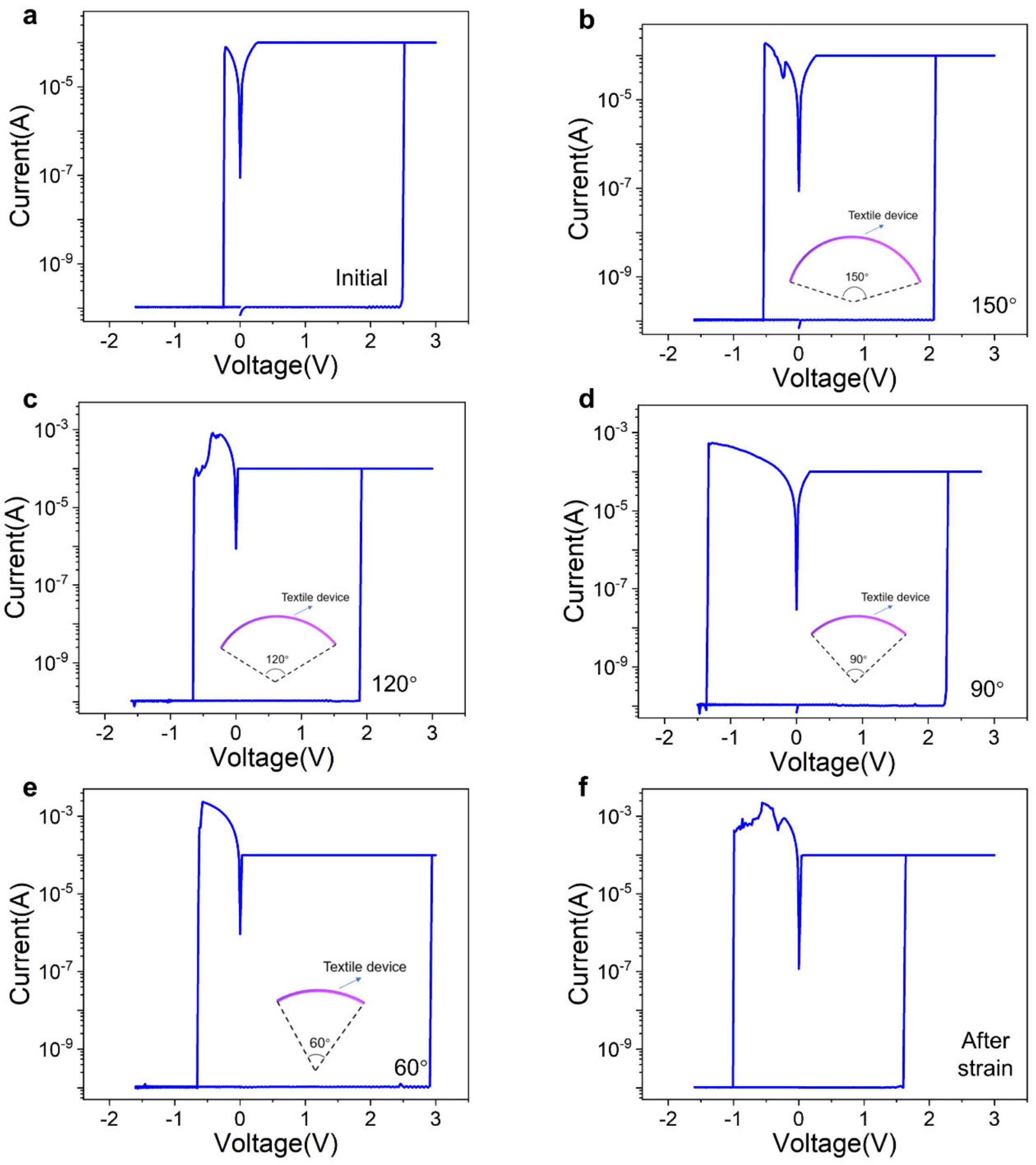

Supplementary Fig. 9 | Resistive switching characteristic of device under different strain measurement. (a) Resistive switching characteristic of device before strain. Resistive switching characteristic of device under strain with bending angle of (b) 150°, (c) 120°, (d) 90° and (e) 60°. (f) Resistive switching characteristic of device after strain.

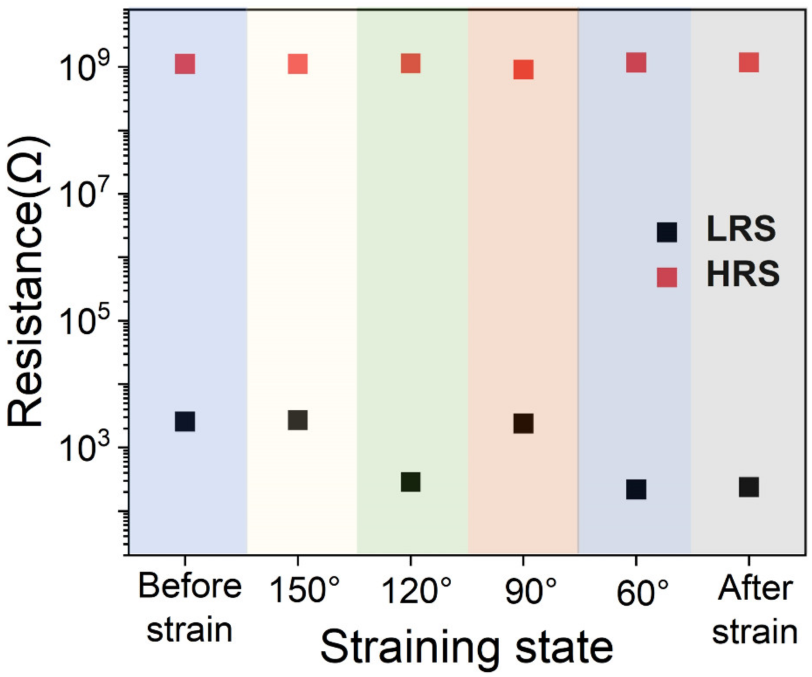

Supplementary Fig. 10 | HRS and LRS of device under different straining state, including before strain, under strain with bending angle of 150°, 120°, 90° and 60°, and after strain. The result indicates the reliable resistive switching characteristic of device.

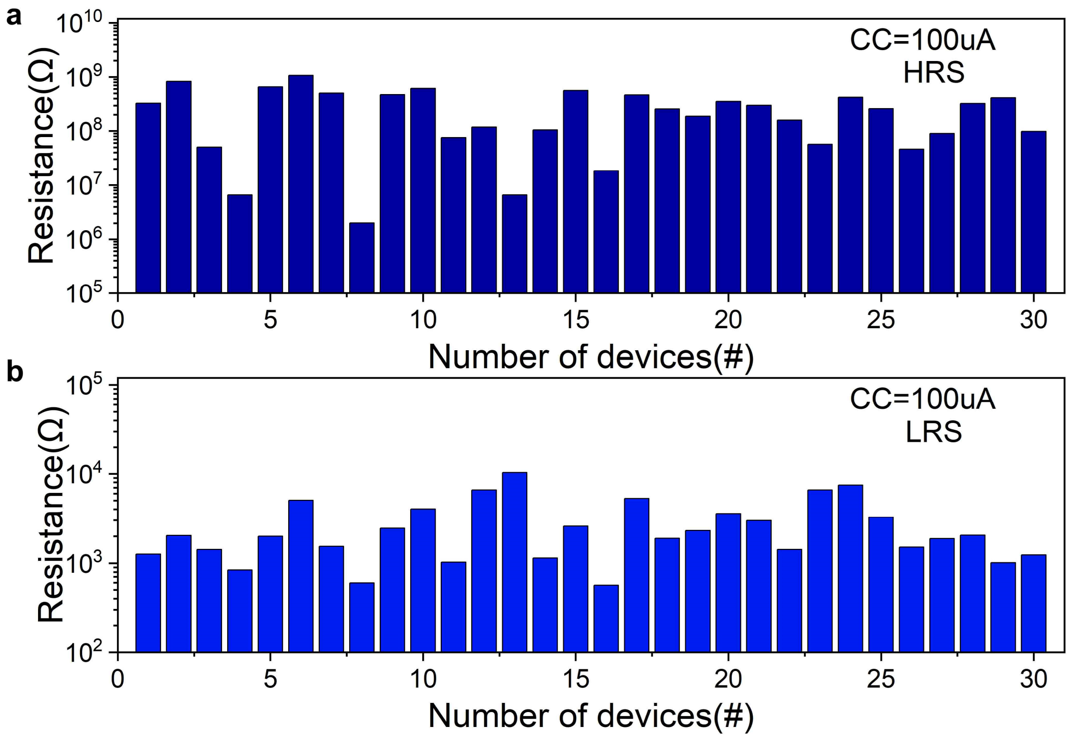

Supplementary Fig. 11 | Statistical results of (a) HRS and (b) LRS extracted from 30 different devices under CC of 100 μA, indicating both high controllability and reproducibility of textile memristors.

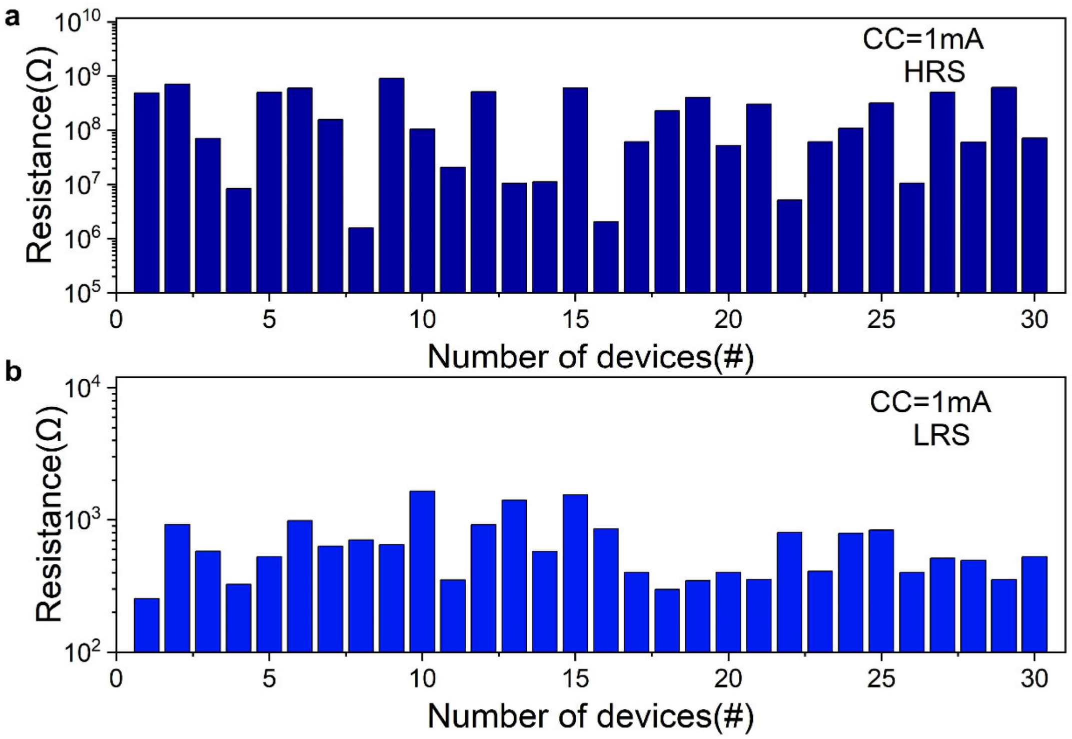

Supplementary Fig. 12 | Statistical results of (a) HRS and (b) LRS extracted from 30 different devices under CC of 1 mA, indicating both high controllability and reproducibility of textile memristors.

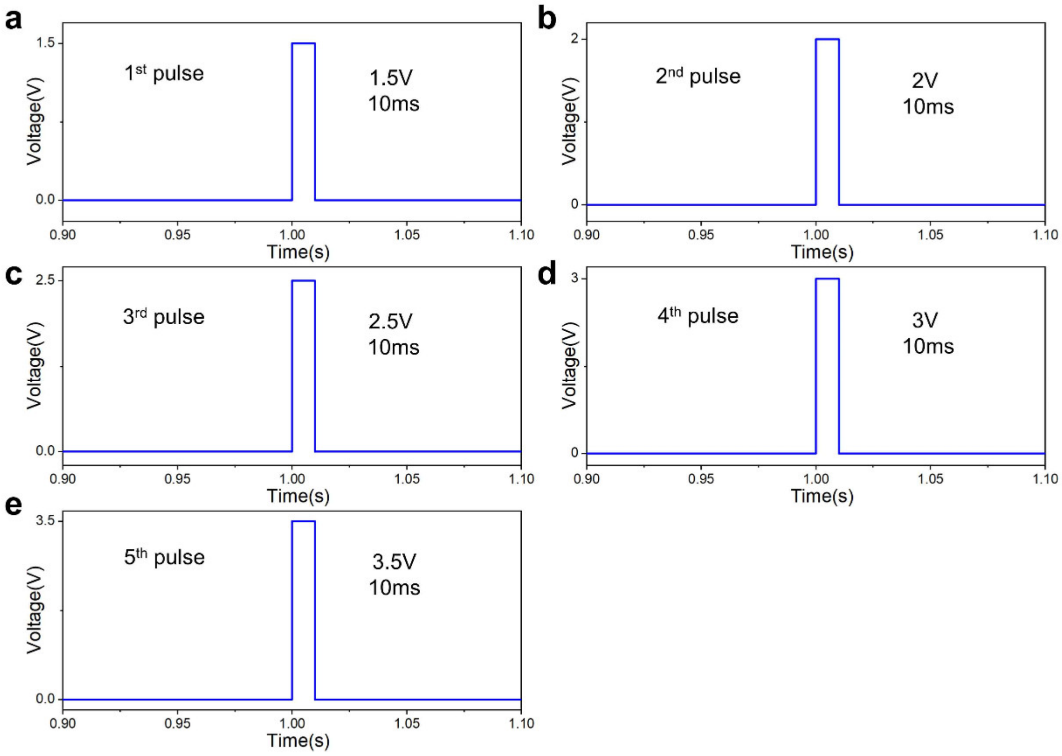

Supplementary Fig. 13 | The pulse waveforms designed for simulating excitatory post-synaptic current behavior, corresponding to Figure 2c in the main text. (a) The first pulse with width of 10 ms and amplitude of 1.5 V. (b) The second pulse with width of 10 ms and amplitude of 2 V. (c) The third pulse with width of 10 ms and amplitude of 2.5 V. (d) The fourth pulse with width of 10 ms and amplitude of 3 V. (e) The fifth pulse with width of 10 ms and amplitude of 3.5 V.

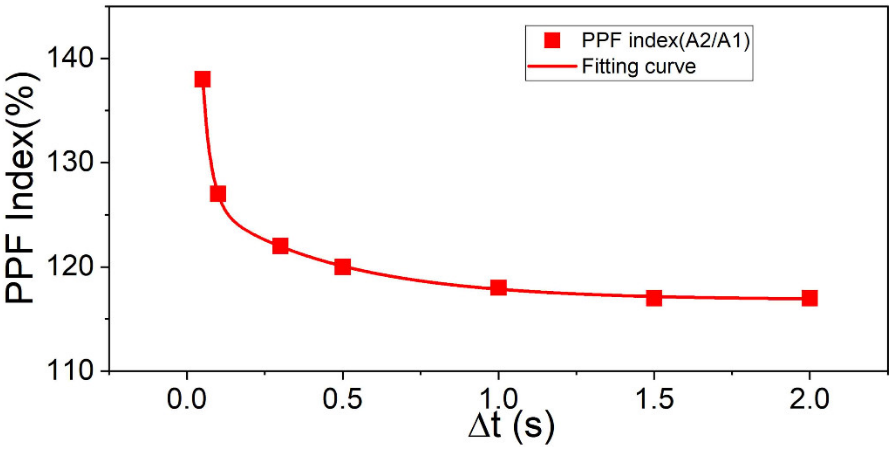

Supplementary Fig. 14 | Paired-pulse facilitation index ($A_2/A_1 \times 100\%$) of artificial synapse fitted by double exponential decay function. The formula is described as[2-4]: $y=A_1\times exp(-\Delta t/t_1)+ A_2\times exp(-\Delta t/t_2)+y_0$, where Δt is the pulse interval time, y_0 is the resting facilitation magnitude, and t_1 and t_2 are relaxation time.

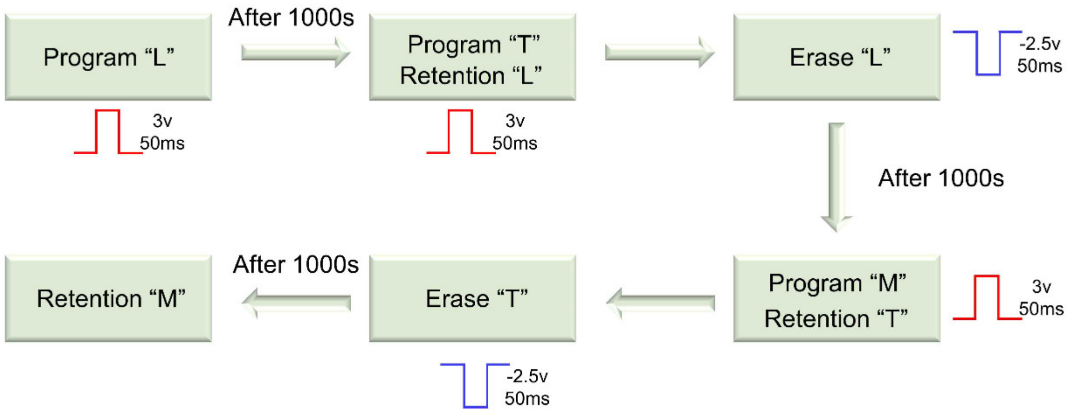

Supplementary Fig. 15 | The flowchart of array-level operations for long-term memory. The letters of "L", "T" and "M" were input to array by applying pulse (3 V, 50 ms) to memristors, which could be erased by negative pulse (–2.5 V, 50 ms). The interval time between different letters is 1000 s, demonstrating the long-term retention characteristics of nonvolatile artificial synapse.

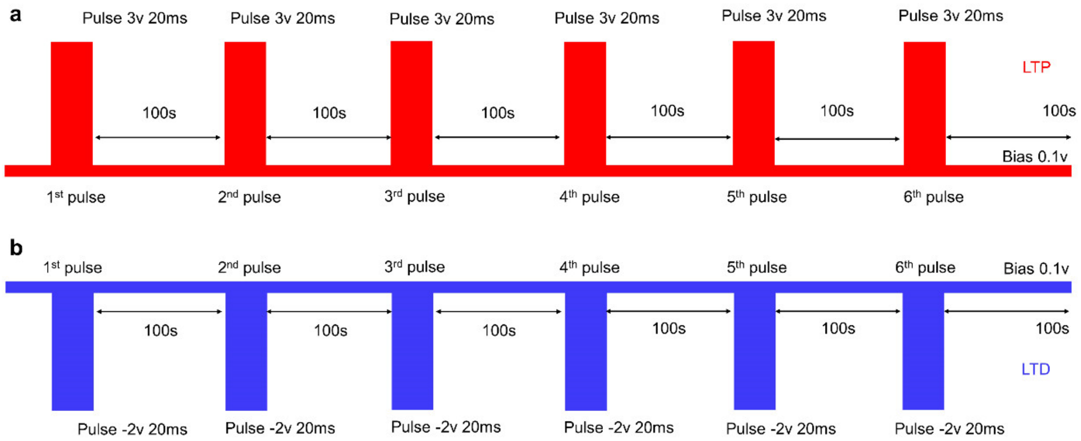

Supplementary Fig. 16 | The designed pulse waveforms of long-term potentiation and long-term depression. (a) During simulating long-term potentiation, the consecutive pulses (pulse width of 20 ms, pulse amplitude of 3 V, pulse period of 100 s) were applied to memristors. (b) During simulating long-term depression, the consecutive pulses (pulse width of 20 ms, pulse amplitude of –2 V, pulse period of 100 s) were applied to memristors.

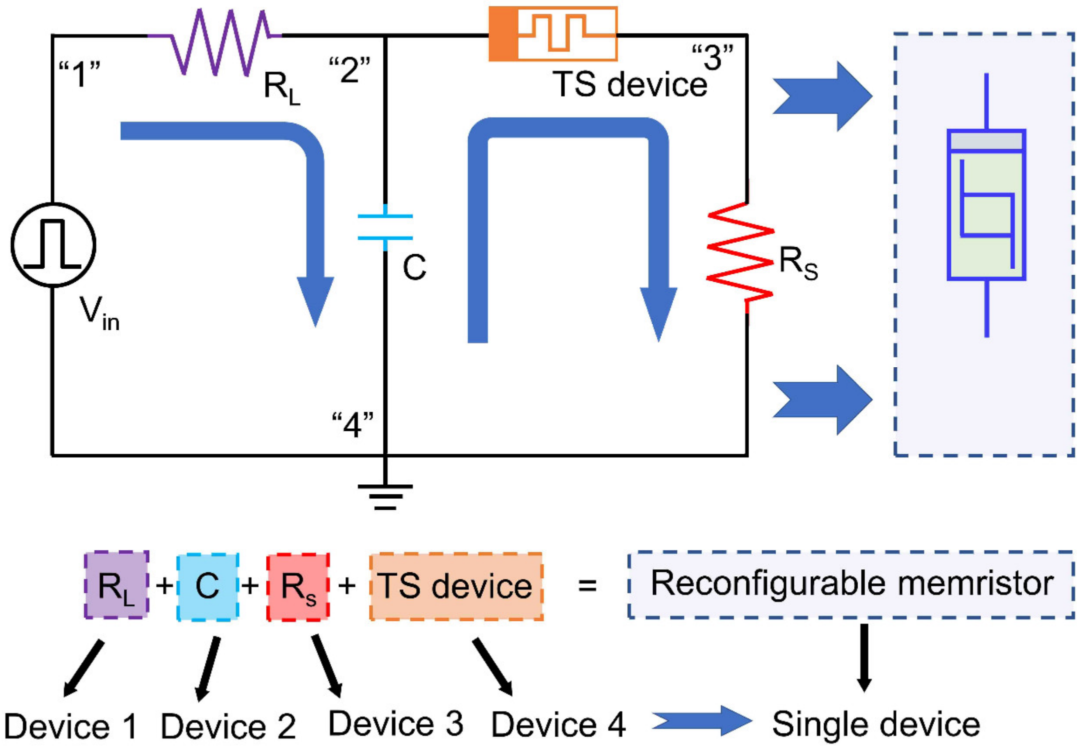

Supplementary Fig. 17 | The comparation of traditional neural circuit and artificial neuron based on Ag/MoS_2/$HfAlO_x$/CNT. The traditional neural circuit consisting of four devices could realize the function of integrate-and-fire as bio-neuron[5]. In this work, single reconfigurable memristor could act as artificial neuron and realize integrate-and-fire function, greatly simplifying the complexity of neural circuit.

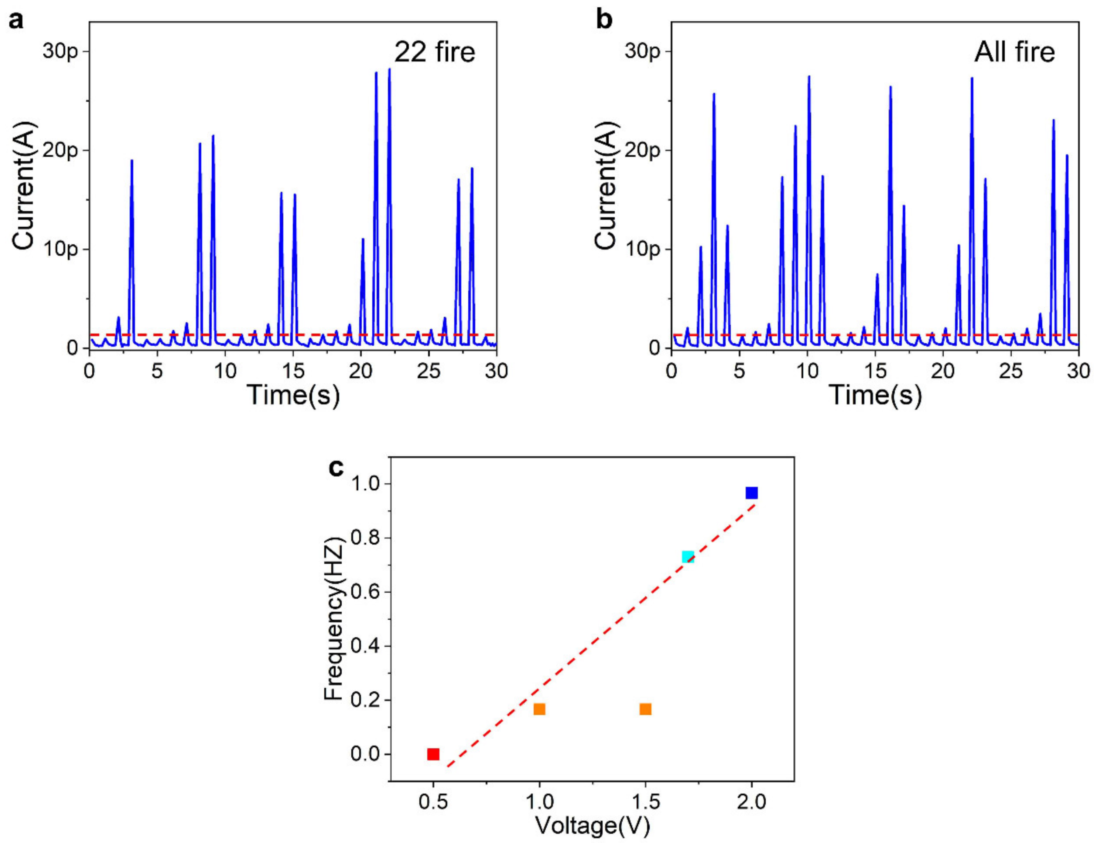

Supplementary Fig. 18 | Strength-modulated frequency response of artificial neuron. (a) Integrate-and-fire function inspired by pulses of 1.7 V, where 22 times firing response were obtained. (b) Integrate-and-fire function inspired by pulses of 2 V, where every pulse could inspire fire. (c) Frequency-voltage curve of artificial neuron, verifying the strength-modulated frequency feature of device.

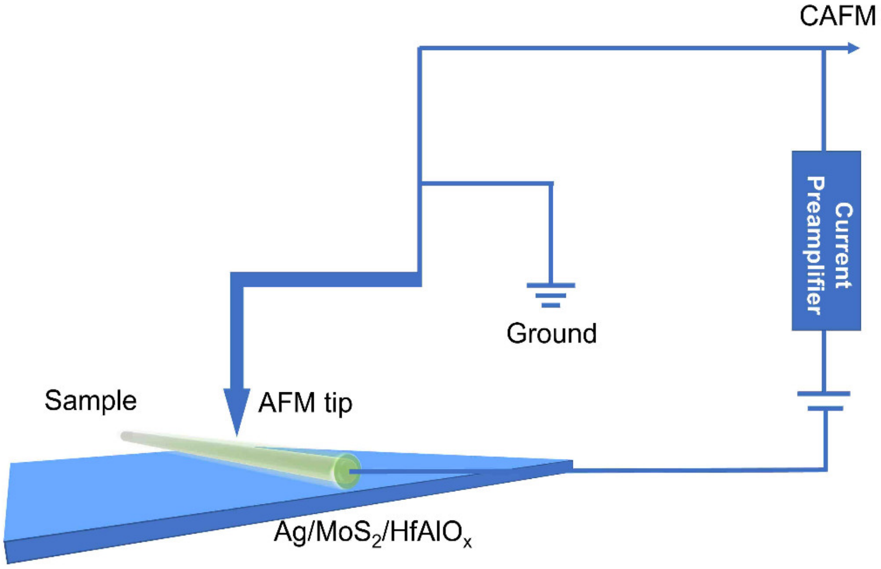

Supplementary Fig. 19 | Schematic illustration of conducting atomic force microscopy (CAFM) for current mapping images, where the AFM tip with bias was connected to surface of Ag/MoS_2/$HfAlO_x$ and Ag fiber was grounded.

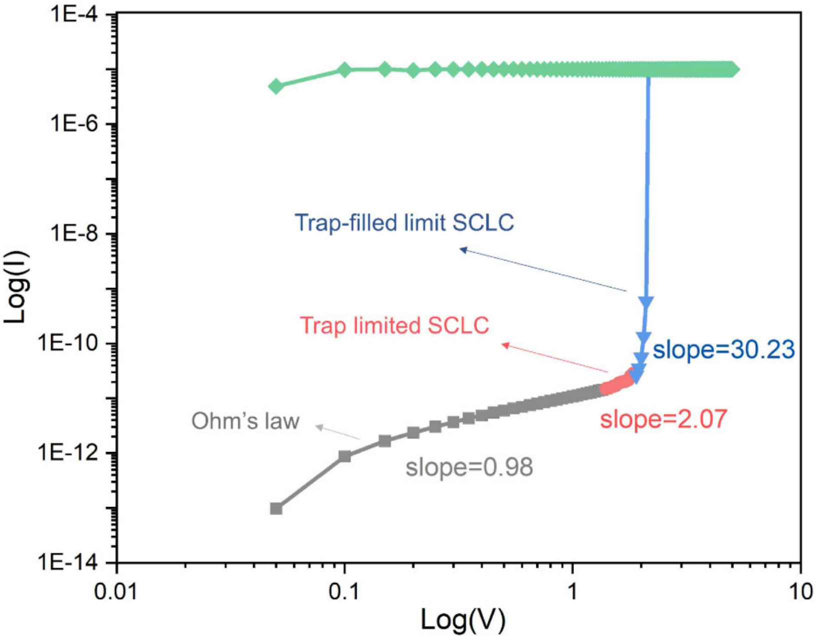

Supplementary Fig. 20 | The positive current–voltage curve of volatile memristor under double logarithmic coordinates, which was divided into three parts by mechanism model fitting[6]. The three parts correspond to Ohm's law ($I \propto V$), trap limited SCLC ($I \propto V^2$) and trap-filled limit SCLC ($I \propto V^n$, $n > 2$). Here SCLC means space charge limited current.

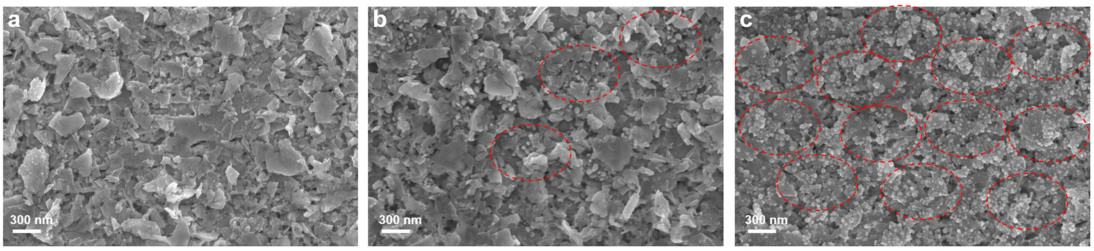

Supplementary Fig. 21 | SEM images of the device interfaces a, before program, b, after a certain degree of program, and c, after a complete program. Scale bar of 300 nm. The area where Ag atoms are distributed is marked with a red circle.

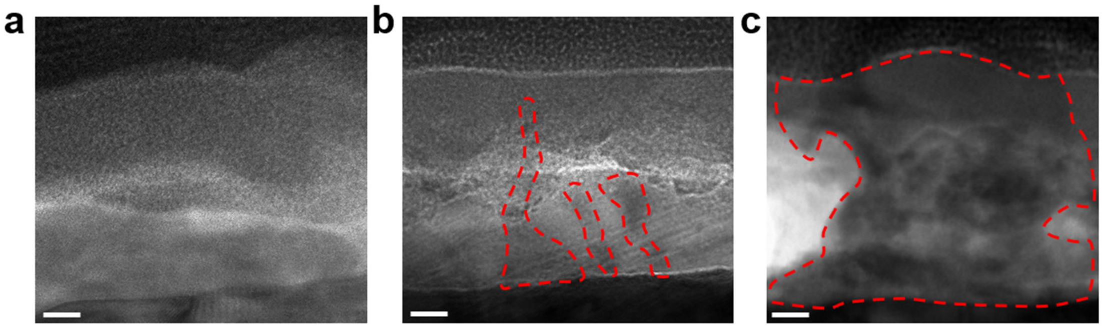

Supplementary Fig. 22 | Cross-sectional TEM images of the device a, under initial state b, under volatile switching state, and c, under nonvolatile switching state. Scale bar of 10 nm. The Ag conductive filaments are marked with a red circle.

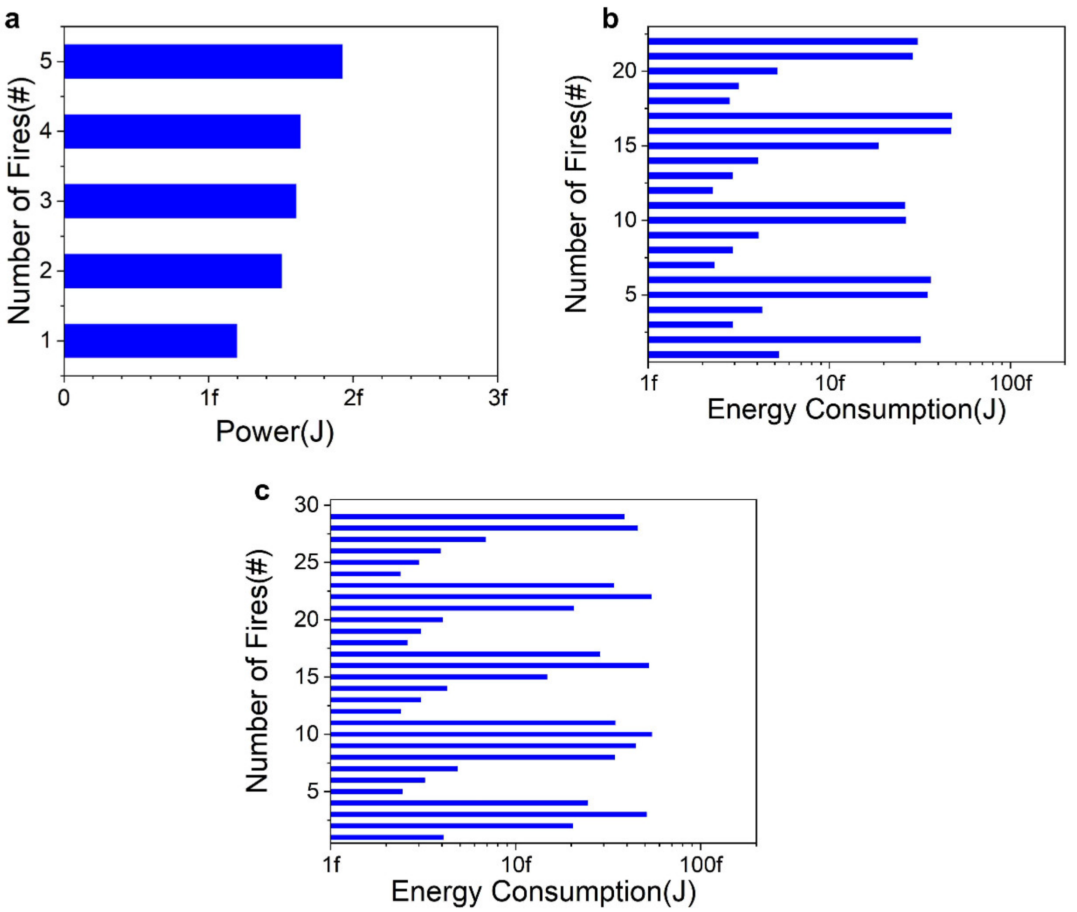

Supplementary Fig. 23 | Statistical energy consumption of artificial neuron under different pulses. (a) Energy consumption of firing operation under pulses of 1 V, corresponding to the response of Figure 3e in the main text. (b) Energy consumption of firing operation under pulses of 1.7 V. (c) Energy consumption of firing operation under pulses of 2 V.

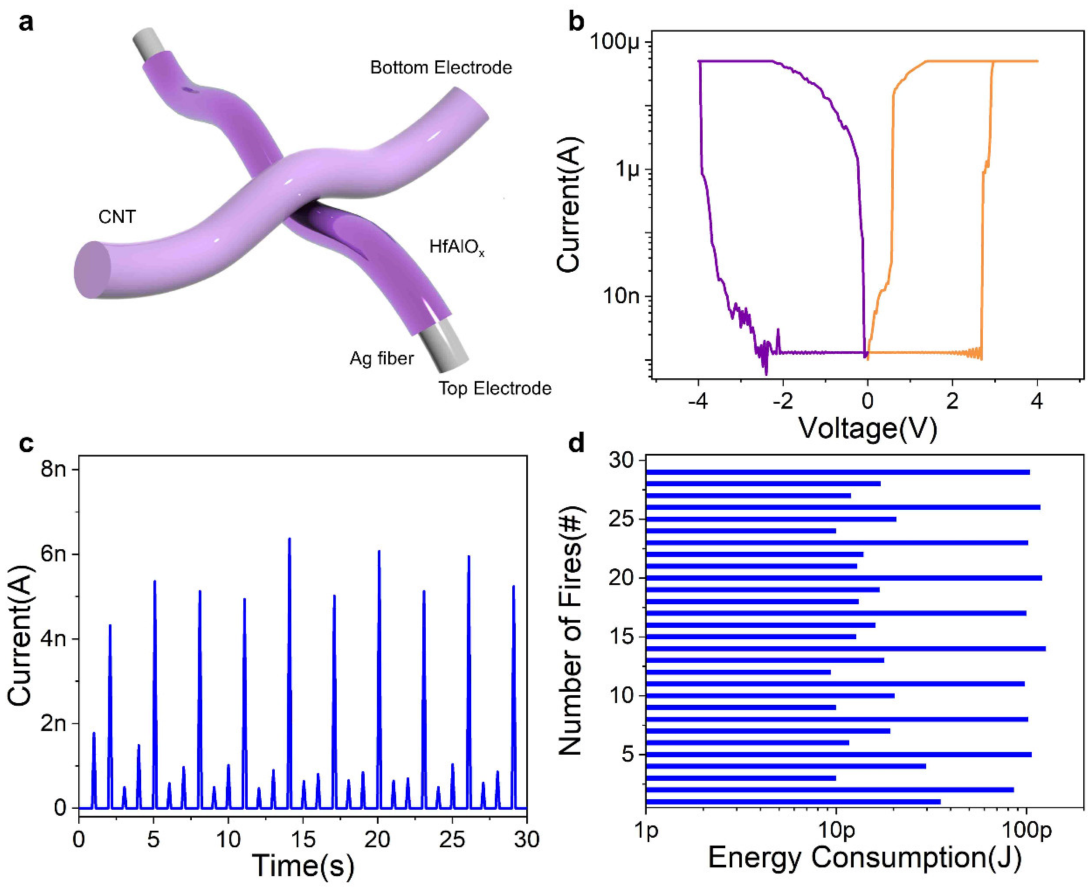

Supplementary Fig. 24 | Control experiment of artificial neuron based on memristor of Ag/$HfAlO_x$/CNT. (a) Structure diagram of Ag/$HfAlO_x$/CNT, where the Ag fiber acts as top electrode and CNT fiber acts as bottom electrode. (b) Volatile threshold switching curve of memristor. (c) Integrate-and-fire function inspired by consecutive pulses, where the threshold value for firing is 1 pA. (d) Energy consumption of memristor during firing, which are all higher than 1 pJ/fire.

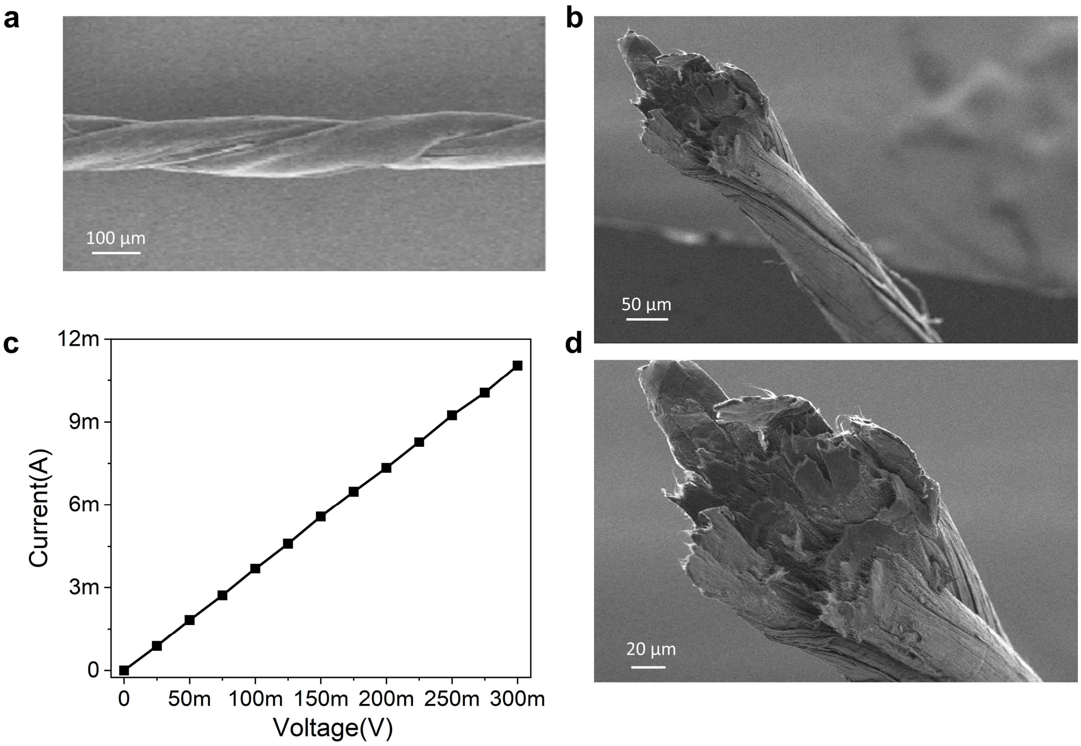

Supplementary Fig. 25 | The heating fiber based on CNT resistor. (a) SEM image of CNT resistor. Scale bar, 100 µm. (b) Cross-sectional SEM image of CNT fiber. Scale bar, 50 µm. (c) Current-voltage curve of CNT fiber used for heating, which is based on formula of $Q=I^2 \times R \times t$. (d) Cross-sectional SEM image of CNT fiber at high magnification. Scale bar of 20 µm.

References

S1. Xu X, Chen J, Cai S, Long Z, Zhang Y, Su L, He S, Tang C, Liu P, Peng H, Fang X. *Adv. Mater.* 2018, 30(43): 1803165.

S2. Meng J, Wang T, Zhu H, Ji L, Bao W, Zhou P, Chen L, Sun Q, Zhang W. *Nano Lett.* 2022, 22(1), 81–89.

S3. Wang T, Meng J, He Z, Chen L, Zhu H, Sun Q, Ding S, Zhou P, Zhang W. *Adv. Sci.* 2020, 7(8): 1903480.

S4. Wang S, Chen C, Yu Z, He Y, Chen X, Wan Q, Shi Y, Zhang W, Zhou H, Wang X, Zhou P. *Adv. Mater.* 2019, 31(3): 1806227.

S5. Zhang X, Wang W, Liu Q, Zhao X, Wei J, Cao R, Yao Z, Zhu X, Zhang F, Lv H, Long S, Liu M. *IEEE Electron Device Lett.* 2017, 39(2): 308-311.

S6. Wang Y, Lv Z, Liao Q, Shan H, Chen J, Zhou Y, Zhou L, Chen X, Roy V, Wang Z, Xu Z, Zeng Y, Han S. *Adv. Mater.* 2018, 30(28): 1800327.
